# Benchtop Technologies for Circulating Tumor Cells Separation Based on Biophysical Properties

**DOI:** 10.1155/2015/239362

**Published:** 2015-04-21

**Authors:** Wan Shi Low, Wan Abu Bakar Wan Abas

**Affiliations:** Department of Biomedical Engineering, University of Malaya, 50603 Kuala Lumpur, Malaysia

## Abstract

Circulating tumor cells (CTCs) are tumor cells that have detached from primary tumor site and are transported via the circulation system. The importance of CTCs as prognostic biomarker is leveraged when multiple studies found that patient with cutoff of 5 CTCs per 7.5 mL blood has poor survival rate. Despite its clinical relevance, the isolation and characterization of CTCs can be quite challenging due to their large morphological variability and the rare presence of CTCs within the blood. Numerous methods have been employed and discussed in the literature for CTCs separation. In this paper, we will focus on label free CTCs isolation methods, in which the biophysical and biomechanical properties of cells (e.g., size, deformability, and electricity) are exploited for CTCs detection. To assess the present state of various isolation methods, key performance metrics such as capture efficiency, cell viability, and throughput will be reported. Finally, we discuss the challenges and future perspectives of CTC isolation technologies.

## 1. Introduction

Cancer is one of the leading causes of death worldwide. According to the International Agency for Research on Cancer (IARC), there are an estimated 8.2 cancer-related deaths in 2012, where 90% of them are caused by metastasis [1]. As a result, metastasis has become the prime prognosis factor in carcinoma patients. Generally, cancer metastasis involves the spread of cancer cells, whereby the tumor cells detach from primary tumor site and be transported via the circulation system to a distant organ to form secondary tumors. These cells, which shed into vasculature, are referred to as circulating tumor cells (CTCs). The presence of CTCs was first discovered by Thomas Ashworth in 1869, after comparing their morphology to tumor cells from different lesions. Despite his discovery, its impact on cancer detection method was less well established in the early stage due to the lack of detailed insight into the mechanisms of tumor.

In clinical practice, the cancer diagnostics are commonly performed through radiological imaging modalities such as traditional radiography (X-ray), magnetic resonance imaging (MRI), computed tomography (CT), positron emission tomography (PET), or ultrasound. These techniques allow visualization of internal body structure. Thus, it enables physicians to delineate the group of tumor cell colonization. However, there are some pitfalls in these techniques. For instance, the deficiencies of resolution in imaging modalities have precluded them to image small numbers of cancer cells before angiogenic switch, which in turn limit the detection sensitivity [2, 3]. Furthermore, most of the cases are normally diagnosed at advanced stages where patients often relapsed within 24 months of therapeutic intervention [4, 5].

In recent years, the emerging data have challenged the traditional theory of metastasis sequential development. In fact, study carried out by Hüsemann et al. highlights that CTCs can be found in patients even before a primary tumor is detected with conventional clinical screening methods [4]. The importance of CTCs is further augmented when there are increasing evidences about the presence of significance correlation between the number of circulating tumor cells and patients survival times. It has been scientifically validated by prospective multicenter studies that patient with cutoff of 5 or more CTCs per 7.5 mL of blood would have poor survival rate [4, 6, 7]. A similar analysis of prognostic value of CTCs among colorectal cancer patient was performed by Allen and El-Deiry. Their study points out that the median progression free survival (PFS) and overall survival rates were twice as high for patient with less than 3 CTCs per 7.5 mL of blood; thus, it has confirmed the previous findings. Additionally, this group also presented significant data which showed that patients with elevated CTCs density after therapy would have poor survival rate [8]. Nevertheless, simple enumeration of CTCs is inadequate because cancer is a constellation of diseases with various pathologic alterations that might cause prognosis. Since the ability in analyzing proliferation of viable CTCs has still been lacking, it is difficult to assess CTC information which is the representative of cellular information available in primary tumor. In fact, the dimension of CTC biological feature is especially significant for basic research pertaining to metastasis as well as drug development. To further complicate matters, the recent appreciation of genetic alterations and biomarker expression, for instance KRAS, within tumors means a single biopsy sample is no longer sufficient [9]. Henceforth, detecting and analyzing these cells on a sample of blood may shed new light on circumvents clinical need to improve therapeutic efficacy as well as the overall patient survival rate. Despite its high potential in cancer treatments, the detection of CTCs from whole blood sample is particularly challenging due to their extremely rare presence, which is only 1 to 10 CTCs per billion normal blood cells in patients with advanced cancer. Besides, the large morphological variability among CTCs has imposed technical challenge to isolate the whole population, where sophisticated algorithms are required to identify the CTCs. Aforesaid, CTC counts are associated with patient prognosis. Thus, a highly sensitive detection method is vital to accurately characterize and enumerate the CTC.

Numerous methods have been employed and discussed in the literature for CTCs separation. To date, the benchtop device developed by Veridex is the sole system approved by United States Food and Drug Administration for the clinical monitoring of CTC counts in patients with metastatic breast, colon, and prostate cancer. Through this approach, the CTCs are distinguished from other blood cells based on their immunoaffinity properties. The selection is carried out by using antibodies coupled with magnetic beads, targeting the tumor-associated antigen on CTCs surface (EpCAM, CK, and CD45). This system is proved to have sensitivity of 87.7% with the capability to detect of approximately 5 CTCs per 7.5 mL of whole blood. Despite the proven clinical ability of this system, a few data have been published, concerning the inconsistently markers expressed by all tumor cells. Noteworthy, EpCAM is not expressed in all histological tumor types and thus might result in false-negative/positive result and limit the purity of the enriched CTC fraction. Using affinity capture method in macroscale analytical system will also lead to permanent attachment of target cells to marker protein on CTC. Consequently, the downstream options for the extraction and subsequent characterization of CTC will be narrowed down.

In addition to immune-affinity based separation, in fact, a large panel of approaches for CTC isolation that are independent of cell surface antigens have been developed on the basis of its physical properties. The majority of the physical methods for CTCs isolation exploit the differences in mechanical and physical properties, including cell size, deformability, electrical polarizability, and magnetic susceptibility. In contrast to the immune-affinity method where epithelial antigens are needed to mediate the intercellular adhesion, this technique is label-free. Therefore, the interference such as sample contamination due to the tagging molecules can be avoided. Since CTCs are unmodified by physical separation, cells isolated using these methods are compatible with wider range of analyses, including those requiring viable cells. Consequently, it allows large range of molecular biological analysis of CTCs to be implemented and thus provides clinician with further insight into CTCs role in tumor formation.

In this paper, we will present an overview about the potential application of benchtop CTC detection device based on their physical properties in clinical oncology. Both detailed description of various separation principle and comparisons based on separation metrics such as efficiency, viability, and throughput are included in this review. Additionally, the challenges faced by each technique will too be succinctly discussed. The readers are encouraged to read the original papers for additional details.

## 2. Comparison of Biophysical and Biomechanical Properties between CTCs and Normal Blood Cells

According to most CTCs histological study, researchers have observed changes in cell cytoskeleton contents and their cytoskeletal structures as it progresses toward a cancerous state. For instance, CTCs are found to have greater nuclear to cytoplasmic ratio, larger size, and distinct nuclear morphology in contrast to the normal cells. In fact, these cytoskeletal changes have resulted in changes in the overall mechanical properties of cells. Computational mathematical model conducted by Rejniak has demonstrated the insight of CTC's deformation trajectories within blood vessel [36]. His finding highlighted that CTCs' cellular biomechanics such as deformability played an important role in metastasis. As an example, in order to invade distal sites, the stiffness of CTCs cytoskeleton is modified subsequently in a very dynamic way so that it could squeeze across small spaces in extracellular matrix and endothelial cell-cell junctions as well as circulating through the small capillaries. In order to successfully perform the metastatic cascade, the CTCs cell membrane must withstand hemodynamic forces and overcome the effects of fluid shears within the blood vessel. Consequently, it alters the conservation of membrane structure, which in turn affects the surface charge (electrical charge), in contrast to normal blood cells [37]. These distinct physical differences in size, deformability, and electrical properties between tumor cells and blood cells have allowed researchers to employ them as physical-based CTC detection method. The physical and biomechanical properties of CTCs will be briefly discussed in the following section.

### 2.1. Size

In morphological studies, the measurement method such as flow cytometry [38], blood smear, or detailed examination with microscopic study are broadly employed to analyze the cell size of CTCs in comparison to normal cells. The size measurement of cell is normally interpreted in cell area, length, width, and shape features. Despite the differences in these measurement methods, they provide similar quantitative value for both CTCs and normal blood cell measurement, respectively. For example, normal human erythrocyte, also called red blood cells (RBC), is found to have biconcave disks with the diameter of approximately 6 to 8 *μ*m [38–40]. White blood cells (WBC) are derived into two groups, which are granulocytes and agranulocytes. The granulocytes such as neutrophils and eosinophils are typically of 12 to 15 *μ*m in diameter [41]. Meanwhile, agranulocytes such as lymphocytes vary widely in size where the small lymphocytes are 7–10 *μ*m while large lymphocytes are approximately 14–20 *μ*m in diameter. Monocytes are among the largest leukocytes with a diameter of 15 to 25 *μ*m in blood smears [38, 41–43]. In contrast to the mentioned blood cells, the size of CTCs reported in the literature is generally larger than normal blood cells, ranging from 17 *μ*m to 52 *μ*m. The clinical relevance of this taxonomy has been investigated and confirmed by many research groups [44–47]. For instance, few groups of study done on breast cancer patients had found that CTCs showed significant variability with consistent elongated shape and most of them were larger compared to the leukocytes surrounding it [45, 48, 49]. Similarly, in a study of patient with prostate cancer, Park et al. had reported that highly ruffled surface membrane of CTCs had created an excess of membrane surface area. As a result, majority of CTCs have larger size in contrast to normal blood cells [50]. The observation of differences in size of CTCs in comparison to other blood cells has motivated several devices to exploit it as their primary label-free separation criteria. Despite the fact that CTCs have larger cells compared to other blood cells, there is a significant overlap in size of CTCs and leukocytes that might hinder size-based separation process. The calculation conducted by Marrinucci et al. had concluded that CTCs are highly heterogeneous including both high and low nuclear to cytoplasmic ratios which might cause them to vary in size [51]. Therefore, proper consideration in the proposed device design and their operational parameters are needed for high efficiency isolation-based-size method.

### 2.2. Deformability

The measurement of tumor cells deformability mainly refers to viscoelasticity, a property of material that falls into a category between that of an elastic solid and a fluid. Cellular viscoelasticity arises from the combination of high water content conflated with a polymerized cytoskeleton structural matrix. In microrheological studies, this mechanical property is governed by cell surface interactions as well as normal force exerted by the cell on the capillary wall under physiological conditions and in response to external signals. Various approaches were employed in vitro to study these mechanical changes, with techniques ranging from conventional atomic force microscopy (AFM) [52–55], micropipette aspiration (MPA) [56, 57], and magnetic twisting cytometry to more recently tailored automated systems such as microfluidic resonator [58] and inertial focusing method [59]. The advantages and drawbacks of these techniques have been discussed in detail [56, 60]. In fact, the tradeoff between experimental automation and complexity of measurable properties method (such as AFM and MPA) has resulted in most measurement lacking throughput and precision.

Despite of their shortcomings, several consistent cell mechanical behaviors are observed across multiple studies where cancer cell lines are employed. Measurements of the cellular mechanical behavior are frequently lumped into a single universal parameter: Young's modulus. It quantifies the measurement of cancer cell stiffness which is intimately linked with the distribution of actin network within cell cytoskeleton. Various studies have compared the viscoelastic properties of cultured cancer cells and blood cells using AFM technique. The stiffness of metastatic cells was generally found to be lesser (Young's modulus of 3.7 kPa–150 kPa) compared to normal blood cells (Young's modulus of 0.2 kPa) [53–55, 61]. This statement is concurred with a recent study conducted by Byun et al. [58], whereby a suspended microfluidic resonator is used to track the cell's velocity as the blood sample spiked with human lung cancer cells traverses through the constriction region of the device. Their result has indicated that lung cancer cells took a shorter time to deform and traveled through the integrated constriction in the microfluidic channel, compared to normal blood cells, thus suggesting that cancer cells exhibit high cytoskeletal deformability [58].

While researching the viscoelasticity properties of tumor cell in the context of metastasis, various studies have expressed the strong correlation between cell deformability and cell malignancy. For instance, Mak and Erickson [56] and Mohammadalipou et al. [57] have measured the mechanical properties of both metastatic and benign breast cancer cell lines with MPA, whereby the metastatic cancer cell lines were found to have longer aspiration length which in turn resulted in higher Young's modulus measurement value (calculated from the pressure slope versus aspiration length graph). Likewise, the AFM-based analysis performed by Chen et al. across prostate cancer cells lines (LNCap-AD, LNCap-AI, and PC-3) indicated that the noncancerous BPH-1 cells were found to have the least elasticity with Young's modulus of 3.7 kPa, whereas the highly metastatic PC-3 cells (Young's modulus of 0.13 kPa) were almost 30% more elastic in contrast to BPH-1. Noteworthy, Young's modulus values described in these experiments were formulated based on the cell elastic properties, using Hertz model, which was used to describe the physical relationship between the applied force and the cantilever deflection on indentation. Apart of cultured cell lines, Chen et al. also have measured Young's moduli of CTCs isolated from blood of patient with castrate-resistant prostate cancer and bone metastasis. When a comparison was made between CTCs and prostate cancer cell line, the values of Young's moduli obtained for CTCs (ranged from 0.23 to 1.1 kPa) were similar to that of PC-3, thus inferring they are metastatic. In accordance with the relationship between cell malignancy and deformability, investigation of ex vivo mechanical changes of cancer cells was conducted by Cross et al. [62]. Their study has reported the stiffness of live metastatic cancer cells taken from pleural fluids of breast cancer patients (Young's modulus of 0.53 ± 0.10 kPa) that was 70% lower, in contrast to the benign cells reactive mesothelial cell (1.97 ± 0.07 kPa) within the same fluid samples. This statement is in agreement with study demonstrated by Chen et al. on prostate cancer cell line, as mentioned previously.

The brief finding presented here indicates that cell deformability can be considered useful parameters to reflect a histological background of a cell and distinguish between nonmetastatic and metastatic cells.

### 2.3. Electrical Properties

Studies of the cell morphology have provided fundamental statement that the cell membrane selective permeability is controlled by electrical charges [37, 63]. The different concentrations of molecules on inner and outer sides of the membrane have created an electrical potential across the membrane. The work by Becker indicated that the major charge carriers of biological cell membrane were negative at physiological pH, such that the healthy living cells were found to have a membrane potential within the range of −60 to −100 mV [64–68]. The negative sign of the membrane potential indicated that the inner surface of the cell membrane is relatively lower than the immediate exterior surface of cell membrane [69]. However, the cell membrane charge was found to be altered during tumorigenesis due to its abnormal metabolic transformation [37]. Therefore, investigation of cell electrical behavior when they are subjected to an electric field can furnish information for the purpose of cancer cell isolation.

In literature, two technique are typically used to measure electrical properties of cells, such as impedance spectroscopy [70] and electrorotation (ROT) [71]. Both methods provide quantitative information of inherent electrical and dielectric properties of cells; such as membrane capacitance, membrane resistance, cytoplasmic conductivity and permittivity. For impedance spectroscopy, it is employed by researches to measure cell suspension dielectric properties as a function of frequency, giving a population averaged value for the cell properties. Meanwhile, measurement for electrorotation is performed on single cell level, which is located at the center of four electrodes. A 90-degree phase excitation signal is applied to the electrode and the cell rotation speed is recorded. Depending on the frequency of ROT excitation signal, the cells will exhibit vary rotation speed based on their cytoplasmic conductivity and permittivity.

Becker et al. has investigated the differences in dielectric properties of metastatic human breast cancer cell line MDA231, erythrocytes and T lymphocytes with ROT method. The result showed that the metastatic human breast cancer cell line MDA231 had higher membrane capacitance (26 ± 4.2 mF/m^2^) than T lymphocytes (11 ± 4.2 mF/m^2^) and erythrocytes (9 ± 0.8 mF/m^2^) [71]. Meanwhile, Qiao et al. presented an investigation of impedance for cell suspensions for four breast cell line, namely MCF-7 (early stage cancer cell), MDA-MB-231 (invasive cancer cells) and MDA-MB-435S (late stage cancer cells), with impedance spectroscopy measurement technique [70]. In their study, Maxwell-Wagner theory was employed to analyze the electrical parameters of a single cell based on the average result obtained from the experiment. The result uncovered that different stages of cancer breast cells can be distinguish by the conductivity presented by each cell. For instance, early stage cancer cell, MCF-7 had higher whole cell conductivity and cytoplasm membrane capacitance (value of 5.58 mS/cm and 3.94 *μ*F/cm^2^) in contrast to late stage of cancer cell, MDA-MN-435s (value of 3.97 mS/cm and 1.10 *μ*F/cm^2^). Based on the outcome of these researches, we are clearly informed that each cell line had a specific electrical signature which could be utilized for identification of cancer cells and differentiation of the pathology stages of malignant cells. The technique which employed the principle of cell's membrane electrical differences for separation is named as electrokinetic (e.g., dielectrophoresis). Its details will be discussed in the next section.

## 3. Benchtop Technologies for CTCs Isolation

In general, the physical cell-based isolation tools that researchers developed for the purpose of benchtop CTCs detection can be classified into two types: the conventional macroscale analytical system and microfluidic devices. For macroscale system, it mainly relies on large laboratory equipment, with the usage of a few milliliters (mL) of cell suspension (e.g., centrifuge). Since these devices often require a large number of cells from human and animal models, it reduces the likelihood for the patient and physician to receive the results quicker, which causes them rarely to be used in point-of-care application. To overcome the limitations imposed by macroscale system, vigorous efforts have been undertaken to develop robust laboratory test over the past decades. As a result gained from the advances of micro- and nanofabrication approaches, there is a growing trend towards carrying out microscale laboratory work, on a scale of one-tenth to one-thousandth of that macroscale system. These miniaturization reaction platforms are termed as microfluidic devices. In contrast to macroscale system, the use of microfluidic device has offered various advantages such as scalable, shorter analysis time and smaller sample size. Furthermore, this technology enables the actuation of fluid and manipulation of bioparticles at microscale. Such feature is especially important for CTCs research in considering the rare presence of CTCs within the blood, as mentioned previously. Both macroscale and microfluidic techniques employed for physical based CTCs detection will be discussed in detail in next section.

### 3.1. Macroscale CTCs Detection System

#### 3.1.1. Density Gradient Centrifugation

**Figure 1:**
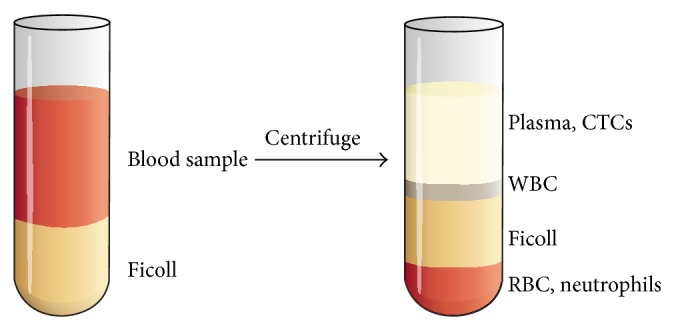
Principle of density centrifugation separation method. Sample is layered on top of a density gradient, Ficoll. Under centrifugal force, particles move through the medium and density gradient and be suspended at a point in which the density of the particles equals the surrounding medium.

Separation of cellular constituents within blood is widely achieved by density gradient centrifugation. This technique uses centrifugal force to separate the cells based on their sedimentation coefficient differences. According to Stokes law of sedimentation, the rate of a particle's sedimentation is directly proportional to its size and density and relative to the density of suspension fluid. As the mixture of diverse cell sample is subjected to centrifugation, the different types of cells will pass through the density gradient at different rates depending on their density, resulting in distinct zones appearance (Figure 1). The heavier particles such as RBC and neutrophils (density of >1.077 g/mL) will appear at the bottom while the CTC, plasma, and mononuclear (density of <1.077 g/mL) will remain at the top as buffy coat [72].

In fact, centrifugation has been employed as early as 1950 by Fawcett et al. to separate cancer cells from peritoneal fluid [73]. A floatation medium is developed in their study to optimize the cell separation into its distinct layer without subjecting the particle to high osmotic or ionic stress. Although this research provided a favorable result such that four layers (which consist of saline, malignant cells, albumin solution, erythrocytes, and leukocytes) are formed in accordance with the particles density, and the use of albumin as floatation medium was costly and uneasy to be prepared [73]. Following this research, another density gradient centrifugation method is set forth by Seal for CTCs isolation in which silicon blending oil was used as floatation solution. In his study, cancer cell is successfully detected in 53% gastrointestinal tract cancer patient samples and 33% breast cancer patient samples [74]. Although both studies showed low separation efficiency, they have uncovered the importance of the use of gradient medium in centrifugation process. Consequently, it has led to escalating efforts in search of gradient media material which provided high separation efficiency. Nowadays, density centrifugation media such as Percoll, Ficoll-Hypaque, and OncoQuick are more widely employed in preclinical and clinical cell centrifugation researches.

Ficoll-Hypaque is a type of sucrose polymer with high synthetic molecular weight. Its gradient media is mainly used in the isolation of human mesenchymal stem cells (hMSCs) [75]. Despite its popularity, various studies reported that this method has low CTCs separation efficiency. Considerable numbers of tumor cells and hMSCs are found to accumulate in the lower fraction instead of preferable upper fraction after density gradient separation. For instance, study conducted by Lara et al. indicated that the use of Ficoll-Hypaque gradient medium had mononuclear cells recovery of 57% [26]. Similarly, when this gradient medium is employed by both van Beem et al. and Aktas et al. for bone marrow mononuclear cell enrichment process, the investigated cell recovery rate is between 15% and 30% [76, 77]. They also highlighted that both cell load and individual harvesting techniques have great impact on the centrifugation performance with Ficoll. Further study done by Ahmadbeigi et al. and Pösel et al. has suggested that such an excess cell loss during Ficoll density gradient centrifugation is a consequence of density medium-related cytotoxicity [75, 78].

Meanwhile, Percoll density gradient media is made of colloidal silica particle suspension. Unlike Ficoll-Hypaque medium which is in ready-to-use form, the osmolality of Percoll medium must be adjusted with saline medium to make it isotonic with physiological salt solution. Such a premade gradient allows it to cover a wide density range for isopycnic banding of all biological particles of interest, such as various cells and microorganism. In the literature, Ellis et al. reported the use of Percoll has obtained more than 90% purity for the gradient that yielded populations of mononuclear cells, neutrophils, and platelets [79]. However, this research is in contradiction with recent study done by Chang et al. on relative isolation efficiencies of both Percoll medium and Ficoll medium. For instance, Ficoll density centrifugation shows high number of isolated mononuclear cells (25.3 ± 8.9 × 10^7^ cells) compared to those with Percoll (13.6 ± 6.6 × 10^7^ cells) [80]. Yet, both Percoll and Ficoll share the same pitfall such that the blood sample tends to mix with the gradient media if the centrifugation did not perform immediately after applying the sample to the gradient media. Such a condition is undesirable as it will cause a reduction of therapeutically relevant CTCs cell populations.

The ongoing study to increase the CTCs recovery rate, focusing on reducing the cell loss and contamination, has led to the OncoQuick centrifugation system. It is a novel technology in which a porous barrier is nestled within the 50 mL centrifuge tube to prevent the lower compartment (separation medium) from mixing with the blood sample, prior to centrifugation. Following buoyant density gradient centrifugation, the cells will be separated and pass through the barrier according to their different buoyant densities. As previously mentioned, RBCs and the granulocytes have higher buoyant densities in contrast to other blood cells. Thus, they are partitioned below the porous barrier. Meanwhile, CTCs along with the mononuclear lymphocytes will remain above the porous layer, which allow them to be easily accessible for subsequent collection and analysis of CTCs. In the literature, OncoQuick has showed a significant improvement in CTCs isolation over centrifugation based on Ficoll and Percoll. For instance, study conducted by Gertler et al. indicated that OncoQuick has higher relative tumor cell enrichment than Ficoll density gradient centrifugation when the separated cell fractions were evaluated with flow cytometry. Despite having low cell fraction (9.5 × 10^4^ mononuclear cells) in contrast to 1.8 × 10^7^ cells by Ficoll, the tumor cell recovery rate is between 70% and 90% for both systems [81]. Similarly, Königsberg et al. have applied the same technique to isolate CTCs from blood sample of 26 metastatic breast cancer patients. The CTCs were spotted in 69.2% of blood samples [27].

### 3.2. Microfluidic Device

#### 3.2.1. Microfiltration

Microfiltration is a technique of flowing cell sample through an array of microscale constrictions in order to capture target cells based on size or a combination of size and cell deformability. Several microfilter designs are developed in the literature for benchtop CTCs separation, varying in terms of their blood passing capability and trapping efficiency. The microfilter developed for the CTCs separation can be categorized based on their geometrical design. Among them, membrane, weir, pillar, and packed bead-based microfilters are frequently discussed in the literature.

**Figure 2:**
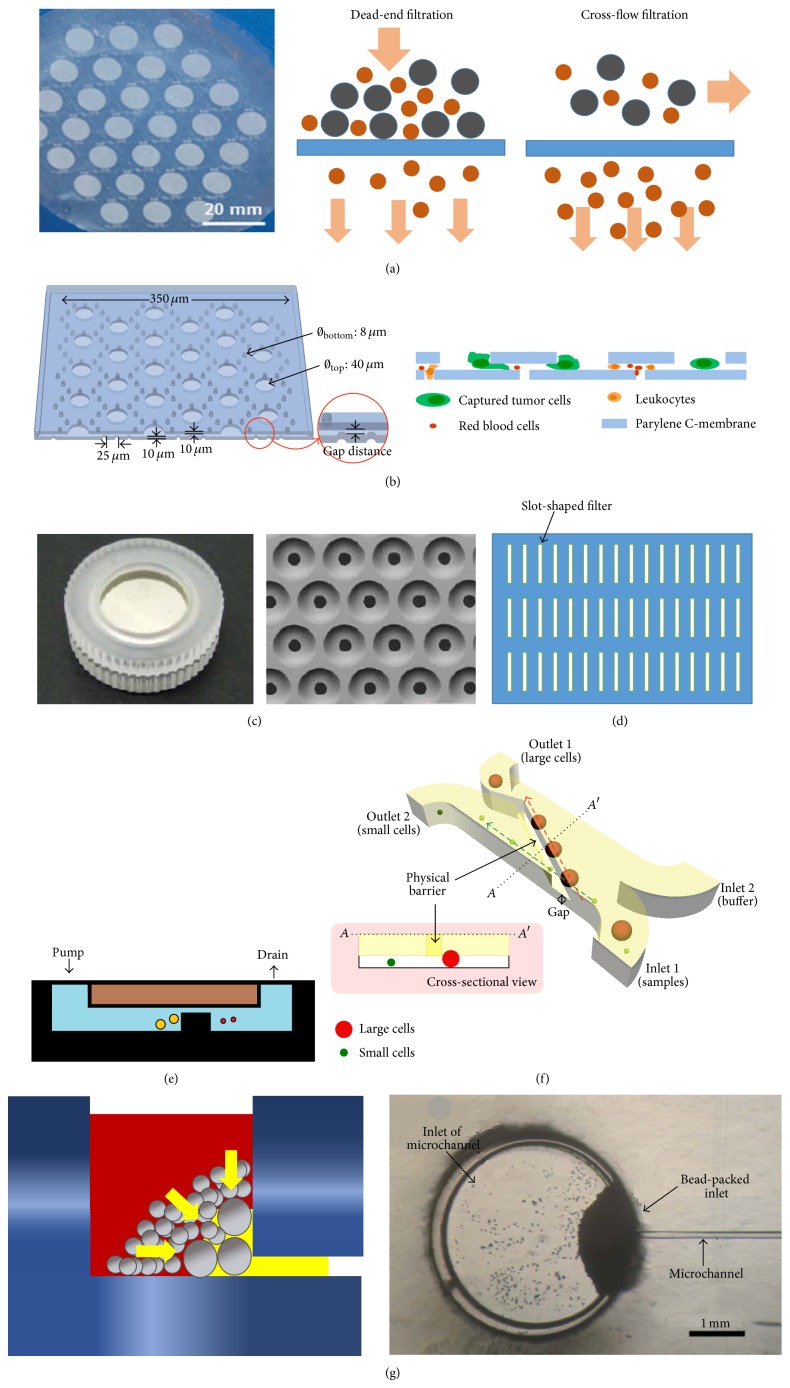
Schematics of various microfiltration mechanisms: (a) membrane microfilter (reproduced with permission from [10], copyright 2014, The Royal Society of Chemistry). Its fluid flow configuration can be further categorized into two types, which is dead-end filtration and crossflow filtration. (b) 3D membrane microfilter with key geometrical parameters labelled. The smaller cells can easily traverse through the gap while the large cells (e.g., tumor cells) will be trapped. Two types of force are exerted in the trapped cell such that force is caused by hydrodynamic pressure from top and supporting force from bottom membrane (reprinted by permission from Macmillan Publishers Ltd.: Scientific Reports [11], copyright 2015). (c) 3D palladium membrane microfilter cassette and its SEM images of filter (reprinted with permission from [12], copyright 2014, PloS One). The cross-sectional view showing tumor cells will be trapped within the gap of the membranes [13]. (d) Membrane slot filter design. (e) Weir-type filter (adapted with permission from [14], copyright 2001, American Chemical Society). A silt-type structure is fabricated within the flow channel to improve the target cells retention. The smaller weir gap is designed to allow human RBC and plasma to pass through while retaining CTCs. (f) Cross-sectional view of diagonally weir-type filtration (reproduced with permission from [15], copyright 2012, John Wiley and Sons). (g) Bead-packed based filtration. The microchannel entrance is blocked by packing large sized beads. Different bead sizes were used to implement a blood/plasma separator at the inlet of the microchannel. Subsequently, when whole blood was dropped into the inlet of the microchannel, the structure was allowed for the capillary flow of blood through the hetero-packed beads. During this movement of blood, the RBC will pass through small pores while big sizes cells such as CTCs will be blocked from flowing into the channel (reproduced with permission from [16], copyright 2012, The Royal Society of Chemistry).

For membrane microfilter, it consists of a semipermeable membrane perforated with a 2D array of small holes (Figure 2(a)). Such filters are commercially available in different pore size and most of the reported membranes for CTCs separation have pore sizes of 6–11 *μ*m diameters. Noteworthy, a pore size around 8 *μ*m in diameter is proved to be optimal for CTCs retention [82]. Since the pore size can be precisely selected at dimensions commensurate with blood cell exclusion, membrane based filtration is suited for microfluidic blood enrichment application. In early research stage, the dead-end flow configuration is commonly employed for microfiltration sample advection such that the blood flow is perpendicular with membrane surface. Particles smaller than the pore sizes will pass through the membrane and vice versa for the larger particle. Despite its simplicity in implementation process, study conducted by Shiau et al. showed that the deposited layer of trapped cells on the membrane in the late filtration period has governed the buildup of filtration resistance. As a result, the efficiency of device to isolate cultured cancer cells from the whole blood sample is reduced [83]. To overcome these issues, a 3D membrane microfilter which consists of two-layer membrane was proposed by Zheng et al. [11]. Both the top and bottom layers have pores defined by microfabrication and the cell capture is realized by the gap between both layers. The important feature of this design is that the larger pores (9 *μ*m diameter) located on the top layer of microfilter patch are aligned with the center of corresponding hexagon pattern of the smaller pores (8 *μ*m diameter) on the bottom later. When the CTC cultures from human blood sample flow through the 3D membrane microfilter, the smaller cell such as RBC and WBC will be easily traversed through the gap; meanwhile, the tumor cells are trapped in the pores of top membrane. The bottom membrane will provide direct force on the trapped tumor cells to counter hydrodynamic force in opposite direction. Subsequently, the concentrated tension stress on cell plasma membrane can be greatly reduced in contrast to the conventional membrane microfilter. This device has demonstrated capture efficiency of 86%, with a throughput of 3.75 mL/min [11]. Very recently, Zhou et al. have reported a new design of 3D microfilter membrane, as illustrated in Figure 2(b) [13]. This device has fundamentally different structure and filtration principle in contrast to Zheng et al. For instance, the pores on the top membranes are five times larger than the pores (8 *μ*m diameter) on the bottom membranes. Consequently, the captured tumor cells will wedge into the gap between the top and bottom of membranes. Since this device features separable 3D membrane, the captured tumor cells from the healthy donor blood sample spiked with multiple cancer cell line can be accessed by separating the two layers membrane. A capture efficiency ranged from 78 to 83% and cell viability of 71 to 74% is reported. Leveraging the advantage of two-layer membrane microfilter, Yusa et al. have developed a palladium filter unit in which a filter cassette (consists of two-layer membrane, with 8 *μ*m sized pores in bottom layer and 30 *μ*m-sized pores in upper layer) is sandwiched in between the upper and lower rings, as shown in Figure 2(c) [12]. In contrast to the previously discussed 3D membrane microfilter in this paper, this device allows only a relatively low flow rate (2.4 mL/min). A further analysis with computational modeling software has indicated an inverse correlation between the numbers of pores with the filter's flow rate. The recovery rate of spike breast and gastrointestinal cancer cells by this 3D palladium device is sufficiently high, which is >85%. Although these devices are reported with high capture efficiency of CTCs, low enrichment factor is showed across multiple studies. To enhance the enrichment factor as well as eliminate the resistance buildup around membrane microfilter, Lu et al. have proposed 2D membrane slot filter, as depicted in Figure 2(d) [28]. Compared to the commonly employed circular pores, slots allow easier deformation of blood cells in their longitudinal direction, which facilitates easier passage of normal blood cells. Furthermore, the large fill factor of cell also reduces the buildup of flow resistance during filtration and thus minimizes the forces exerted on cells. Consequently, a high viability (>90%) and high recovery rate (>90%) of isolated cancer cells are reported. Apart of changing the architectural design of membrane microfilter, a crossflow configuration is introduced to resolve the problems of pressure buildup during membrane filtration (see Figure 2(a)). Unlike dead-end flow configuration, its filtered flow is parallel to the filtration surface. Membrane microfilter developed by Adams et al. using this principle has demonstrated the ability to capture more than 98% of MCF-7 cancer cells from a diluted 7.5 mL blood sample, given that the membrane consists of uniform patterned distribution of >160000 pores with diameter of 7 *μ*m [10].

Apart of membrane based microfiltration, weir-type structures are typically employed as filter element across the width of the microfiltration chip. It involves an individual barrier obstructing the flow path to trap most of the CTCs from the blood sample while WBC and RBC will pass through the narrow slit located on the top of barrier as shown in Figure 2(e). In the literature, such a device is commonly used to isolate white blood cells from the whole blood sample [84–86]. Up to the moment, Chung et al. are the first and only group which employed weir-based microfiltration to perform CTCs isolation from the unprocessed whole blood cells. Their proposed weir-type device featured a barrier with height of 10 *μ*m and 80 *μ*m in width across the main channel at a small angle of 10° (Figure 2(f)), which effectively allowed the passage of 99% of blood cells (erythrocytes, 6–8 *μ*m; leukocytes, 8–10 *μ*m) that passed through the barrier while impeding the CTCs (>10 *μ*m). This strategy is found to achieve high enrichment ratios (>2 × 10^4^) and recovery rates (>95%) at the flow rate of 20 mL·hr^−1^ [15]. In contrast to weir-type structure, the pillar structures are much more widely used in CTCs isolation [29, 87–89]. Similarly, to the weir-based microfilter, the layout of this device consists of arrays of pillar structures within the main flow channel to allow cells smaller than the slit to pass through it. Using this technology, Lin et al. (2010) have demonstrated recovery rate of >90% in 57 blood samples from cancer patients [29].

Besides, the performances of bead-packed based filtration for CTCs isolation process too are discussed in the literature. Such a method is typically incorporated with the capillary-driven analysis systems, whereby a batch of uniform (diameter of 45 *μ*m) [30] and nonuniform beads (diameter ranged from 100 *μ*m to 15 *μ*m) [16] are packed into a chamber and act as the filter element (Figure 2(g)). Subsequently, when the blood is channeled into the filtration chamber inlet, blood cells such as RBC and WBC are allowed to flow through the packed-bead whereas CTCs will be immobilized within the packed bed. To prevent the cells from binding to the beads, protein-blocking solution is sequentially introduced before the experiment to develop hydrophobicity on the channel and bead surface. According to study conducted by Arya et al., the overall CTCs' capture efficiency for packed-bed based on polystyrene and chitosan beads was lower (varied between 21% and 40%) in contrast to filtration generated by membrane, pillar, and weir-based structure.

In general, the main advantages of microfiltration method lie in its simplicity and its capability to obtain the fractionation of whole blood in a single pass. Additionally, it allows for the counting of CTC per milliliter of blood and maintains cell integrity for CTC detection and further characterization. To achieve high purity and recovery rate with this device, two parameters such as the flow rate of the fluid and the cross section of the pores or the gap dimensions require proper consideration. The fluid flow rate will impact the force applied to each cell as it is deformed through a constriction, whereas the pores or gap dimensions determine the size and deformability of target cells that can be captured by the filter. The significance of their role in filtration is further highlighted when mathematical study conducted by Ma et al. discovered the direct correlation between cell lysing and the applied mechanical trauma (e.g., shear force), such that membrane area with increment exceeding 3% will result in permanent damage on the cells [90]. Since the effectiveness of the isolation mechanism is controlled by the tailored gap and pores dimensions, a few problems have aroused. Firstly, the mechanical filtration system which separates cells based on microfilter's geometrical differences is not universal. Variation in microfilter architecture is required when the size of target cells is changed. Besides, the heterogeneity of CTCs has caused the smaller cells which exhibit carcinoma characteristic from being detected by this device. Consequently, a loss in the target cells for further downstream analysis is feasible. Moreover, the high concentration of blood cells can easily cause clogging of microscale constriction or filter structure designed for cell sorting in microfluidic devices, compromising their performance for applications involving whole blood samples. Noteworthy, CTCs are needed to be removed from membrane microfilter to perform downstream analysis (such as PCR-assays and cytomorphology). However, CTC handling such as aspiration and ejection of single cells into the PCR tube by micromanipulator is time consuming. Furthermore, the nontransparent material of certain membrane microfilter has resulted in the need to use upright fluorescence microscopy rather than the usual inverted fluorescence microscopy for manipulation of CTC.

#### 3.2.2. Acoustophoresis

**Figure 3:**
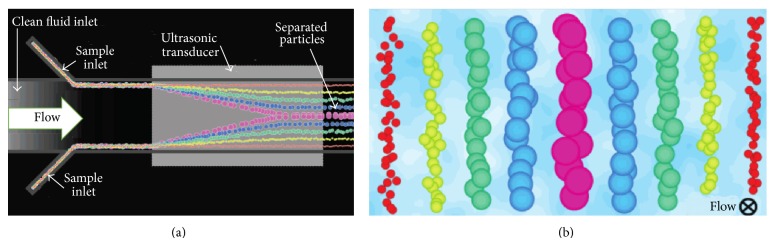
Illustration of (a) an acoustophoresis device and (b) the particle gradient within the microchannel cross section after passing over the transducer (adapted with permission from [17], copyright 2007, American Chemical Society).

Acoustophoresis is the separation of particle using high intensity acoustic waves. Its device consists of interdigitated piezoelectric transducers, which are employed to generate acoustic standing wave. When the cell suspension is subjected to the acoustic field, cells will experience acoustic force. Depending on the cell density and the difference of compressibility properties between cell and the surrounding fluid, this force can vary by orders of magnitude which in turn translates cells toward the pressure nodes (point where the periodic pressure variations are zero) or antipressure node (point with maximum acoustic pressure). If the flow rate, acoustic force, and particle mixture are correctly balanced, particle gradient will be developed across the channel at its end as illustrated in Figure 3. By introducing multiple outlet branches into the microfluidic design, the cells can be separated and guided toward their respective outlet.

Following the acoustophoresis success in RBC sorting by Petersson et al. [91], the differing of acoustophysical properties of CTCs is investigated as the principle for separation from other blood cells. Up to the moment, there are only two groups that have published their work on the performance of acoustophoresis in preclinically CTCs separation. A first effort to capitalize on this aspect was reported by Augustsson's group. They have presented an acoustophoresis device with trifurcation inlet and outlet to separate three different prostate cancer cell lines (DU145, PC3, and LNCaP) from no-cancer control subjects' blood sample spiked with the mentioned cancer cells [32]. As previously mentioned, cancer cells have higher density and compressibility in contrast to normal cells. Consequently, they are found to move toward and align themselves in the center of the channel (pressure node), while normal blood cells can be detected near the channel wall (antipressure node). To minimize the influence of parabolic flow profile, which may otherwise affect the efficacy of tumor cells separation from WBC, method of cell prealignment on paraformaldehyde (PFA) fixed cells before acoustophoretic separation is introduced into their device architecture. In contrast to typical nonprealignment method [17], an improvement in cell separation is observed, yielding tumor cells recoveries of 87 and 83% of DU145 and PC3 cells, respectively, with regard to WBC suppression of 99.3%. When the device performance on nonfixed sample is evaluated, changes in intrinsic acoustic properties are experienced by the WBC such that the WBCs are detected to have high depletion rate at low acoustic energy. However, the tumor capture efficiency is virtually unchanged in contrast to PFA fixation sample; that is, the device shows an increase in separation efficiency for both samples in accordance with acoustic energy. For instance, the average central outlet recovery of DU145 cells (nonfixed sample) was 85.4% and 96.6% at *E*_ac_ of 120 J/m^3^ and 188 J/m^3^, respectively. Meanwhile, for nonfixed method, the average DU145 cell recovery was 36.1% at *E*_ac_ of 66 J/m^3^ and 83.7% at *E*_ac_ of 103 J/m^3^. Additionally, this research also highlights that no significant difference in cell viability between acoustophoresis treated and untreated cells was found for both types of sample. This statement is concordant with study done by Burguillos et al. [33], focusing on the effect of acoustophoretic force on prostate tumor cell's viability and proliferation. Changes in mitochondrial potential as well as cell function via their inflammatory response are selected as the measurement parameter for cell viability. Their experimental result depicted no changes in mitochondrial potential due to acoustophoresis. Also, the cell properties are unaltered after acoustophoretic processing as measured by cell turnover assays as well as inflammatory cytokine response up to 48 hours following acoustophoresis [33].

Although both studies suggested that the microchannel acoustophoresis can be used for effective CTC's detection, this technology is still under preclinical development stage. The main challenge for acoustophoretic microfluidic device to be clinically relevant is that the current system is unable to generate data for blood samples containing much lower concentrations of cancer cells. Furthermore, in accordance with most microfluidic systems that handle particles, acoustophoresis systems are also challenged by clogged systems and occluded channels when the cell concentration is increased. Such a condition can be countered by extending the length of chip, the optimization of flow rate within the microchannel, and the increase of the acoustic force. Although the increase of acoustic force can result in significant improvement of tumor cell recovery which is low in concentrations, Petersson et al. [17] have reported that the acoustic force cannot be increased without limit as high acoustic pressure can cause biological cells lysis. Henceforth, there is a need for acoustic radiation force model which can be used to calculate the force magnitude to be modified to accurately represent the setting in a microfluidic chamber.

#### 3.2.3. Dielectrophoresis

Dielectrophoresis (DEP) is one of the phenomena commonly grouped as part of AC electrokinetic. It refers to the particles motion under nonuniform electric field. Depending on the conductivity and permeability of the cells as well as its suspending medium, the cells can exhibit attractive and repulsive response at a given electric field frequency. For instance, if the particle is more polarizable than its surrounding medium, the particle will experience a net force toward the high electrical field gradient. Such a condition is termed as positive-DEP (pDEP). Meanwhile, negative-DEP (nDEP) happens when the surrounding medium is more polarizable than the particle which causes the particle to move toward the low electrical field region. As alluded in previous section, CTCs feature high surface areas which in turn give them larger capacitance per unit area in contrast to the normal cells. These factors have contributed to the differences in cell dielectric phenotypes, thus directly affecting their motion under electric field in contrast to the normal cells. The carcinoma cells will exhibit nDEP and vice versa to the normal cells. In the literature, the benchtop DEP device has been successfully used to isolate oral cancer [92, 93], colon cancer [94], breast cancer [31], lung cancer, and prostate cancer cells [95], with a recovery rate of 70–90%. Following these successful separation and subsequent preliminary DEP analyses of cancer cells, a multitude of DEP-based clinical CTCs isolation studies are ensued.

**Figure 4:**
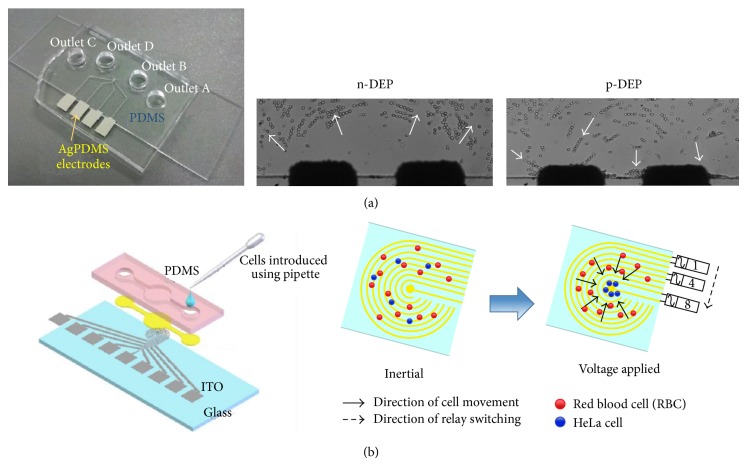
Schematic of DEP cell isolation devices. (a) DEP microchip with 3D side wall microelectrode. By imposing an AC voltage on the side wall microelectrodes, cells will experience repulsive (nDEP) or attractive forces (pDEP), depending on their relative polarizability between cells and fluid (adapted with permission from [18], Copyright 2011, American Chemical Society). (b) DEP system with contactless microelectrode. This method is capable of manipulating cells without direct contact between electrodes and sample. The schematic of cDEP platform design is showed, such that the electrode is inserted into two conductive microchambers, and is separated from the microfluidic chamber by thin insulating barriers. Consequently, cell adherance to the microchip can be prevented. To accumulate the target cell onto the central microelectrode, a stepping electric field is generated such that the applied electric field is subsequently switched between the adjacent electrode pair via relays. Cell which experienced pDEP will be guided along the direction of stepping electric field toward the center of circular electrode (adapted with permission from [19], copyright 2012, Journal of Medical and Biological Engineering).

In order to convey the proper control of DEP force on blood sample, it is necessary for researchers to select or develop appropriate microelectrode designs which fix their studies requirements. For the existing DEP devices, the 2D metallic microelectrodes with various geometries, such as interdigitated [96, 97], castellated [98], curved [31, 99], spiral [100], and ring shape [101], are normally patterned within the chamber of microfluidic device to generate nonuniform electric field that is of desirable strength for the CTCs isolation. These microelectrode designs are easily to be fabricated with common lithograph technique. Nevertheless, they are compatible with on-chip analysis for continuous manipulation of blood samples. However, there is a pitfall in this technique such that electrical fields are found to decay exponentially with the distance away from the electrode. It has caused cancer cells away from the electrode less controlled by DEP force. To circumvent these issues, Lewpiriyawong et al. have suggested the use of 3D electrode deposited at the channel wall to generate electrical field covering the whole volume of channel, as illustrated in Figure 4(a) [18]. This configuration has been successfully demonstrated for cell sorting in study done by Wang et al. (2009) [102]. Their results show an improvement of 15% in cell recovery rate in contrast to the conventional microelectrode. However, due to its fabrication complexity, such a configuration is yet to be tested with CTCs. In the latest research, Huang et al. developed a DEP-based technology known as contactless DEP (cDEP) which replaced the metallic electrodes by fluidic electrode channels (see Figure 4(b)) [19]. The cDEP is capitalized on the sensitivity of traditional DEP, while eliminating challenges such as bubble formation, electrode delamination, expensive fabrication, and electrode sample contamination. The experimental results have indicated that the cervical carcinoma cells are successfully isolated from the concentrated RBC with a recovery rate of 64.5%.

The major advantage of DEP compared to other separation schemes is that the variability in the frequency response of cells is selective enough for DEP microsystems to monitor therapeutic efficacy and to account for constantly evolving tumor phenotypes. However, some consideration might need to be taken into account during the design of DEP device for blood sample. In practice, most of the reported on-chip DEP separation microfluidic devices require the use of low conductivity medium in order to generate pDEP force to trap cell of interest. Noteworthy, blood is a very high conductivity medium. As a result, it might cause cells to experience nDEP most of the time and thus influence separation performance, thus influencing the cell separation performance as well as the purity output. Though Gascoyne et al. [96] suggested that CTCs enrichment through blood dilution allows DEP devices to have optimal recovery, Leu and Liao [103] have reported that the actual extraction efficiency drops for 20% if the dilute ratio 1 : 3 of whole blood sample was conducted. Despite the use of nDEP that is able to levitate particles above the electrodes and thus protects vulnerable biological particles from high electric fields, RBC could also be irreversibly damaged as a means of cell rupturing if electric field much higher than 0.12 MV/m is applied [104].

#### 3.2.4. Hydrodynamic Sorting

Hydrodynamics is one of the important forces that govern the behaviour of microfluidic devices. Its fundamental concept is represented with Navier-Stokes equation, which describes the motion of fluid substances at a given point in space and time. Since the build-in-channels of microfluidic devices have the dimension of less than 1 mm, the flow generated within them is completely laminar at Reynolds number below 2000. Reynolds number (Re) is a dimensionless parameter representing the inertial to viscous forces ratio in a flow. At this low Reynolds number, a particle has been expected to follow fluid streamline, superposed with its intrinsic Brownian motion due to their differences in size and density [105]. By resorting to this principle into the designated microdevice geometries, the cell can be separated according to the different flow rates of parallel fluid flow into the desired outlet. Such a technique is termed as hydrodynamic sorting and it can be further classified into 3 types, which are pinched flow fractionation, deterministic lateral displacement, and inertia separation.

**Figure 5:**
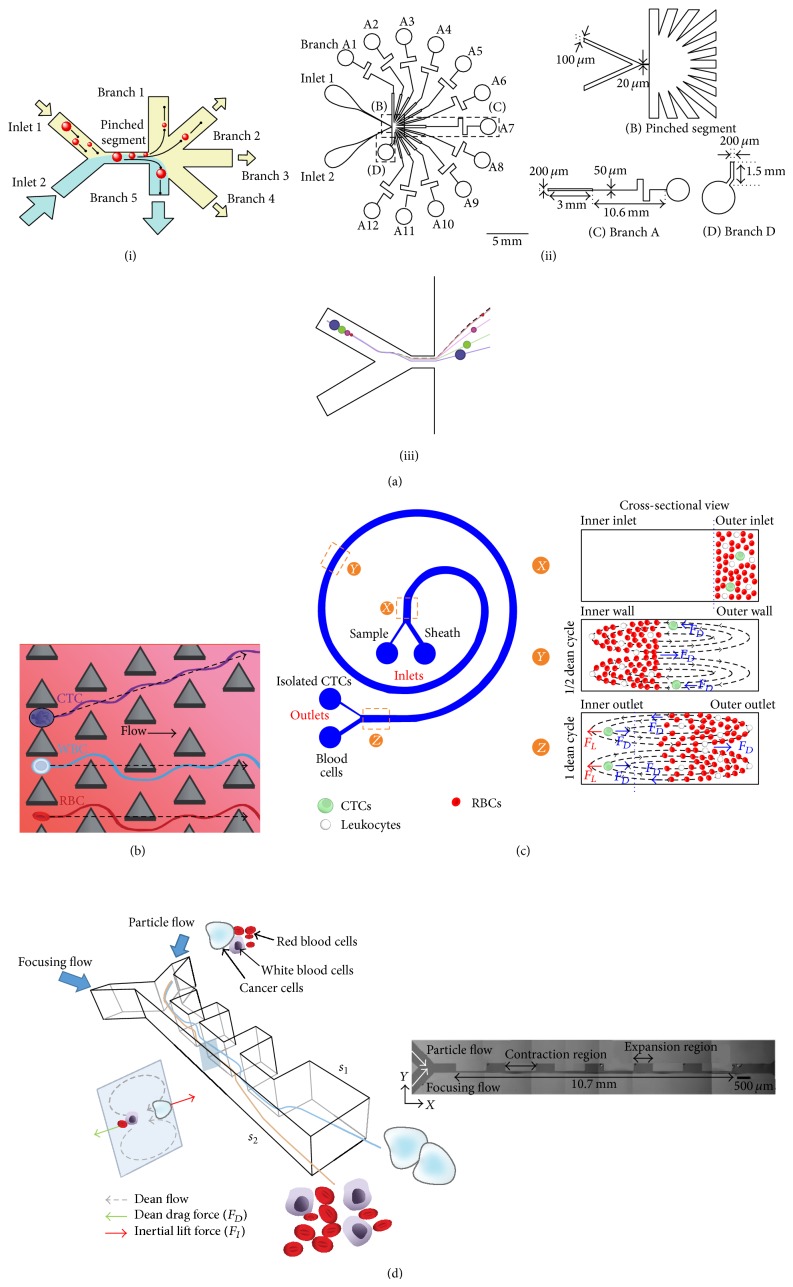
Types of hydrodynamic cell sorting: (a) Pinched flow fractionation. In both microfluidic design by (i) and (ii) Takagi et al. [20], multiple branch channels with different channel dimensions were arranged at the end of the pinched segment, thus resulted flow rate distribution to each channel was different. Cell would then enter into their respectively outlet in accordance to their size (Reprinted with permission from [20]. Copyright 2005 The Royal Society of Chemistry.). As illustrated in (iii) [21], the smaller particle will closely follow the fluid streamline and move toward the upper portion of the exit area, while the larger particles move closer to the center. This is due to the smaller cells tends to move faster under hydrodynamic force which resulted them to press closer to the wall as the flow ratio increase (Reprinted with permission from [21]. Copyright 2013, John Wiley and Sons.). (b) Deterministic lateral displacement. The presence of array of microposts (which each row of posts is slightly offset laterally with respect to the previous row) will cause the cells below critical hydrodynamic diameters (such as WBC and RBC) to follow the streamlines cyclically through the gaps. Meanwhile, cell above critical hydrodynamic posts such as CTCs will be moved by lateral drag into sequential streamline at each post (reprinted with permission from [22], copyright 2013, AIP Publishing LLC). (c) Inertial separation. When the blood sample is pumped into the spiral channel, the centrifugal acceleration of fluid flow will result in the formation of two symmetrical counter-rotating vortices across the channel. The smaller cell such as RBC and WBC will move along the vortices toward the inner wall and back to the outer wall, while larger CTCs will focus along the inner wall due to the additional strong inertial lift forces (reprinted by permission from Macmillan Publishers Ltd.: Scientific Reports [23], copyright 2013). (d) Contraction expansion microfluidic device. Dean drag forces are induced at the entrance of contraction region and thus result in blood sample which flow through this region to have an influence by inertial lift force. RBC and WBC will move toward *s*_2_ while cancer cells move toward *s*_1_ (reprinted with permission from [24], copyright 2013, American Chemical Society).

Pinched flow fractionation acts according to the Zweifach-Fung effect, such that the blood cells are separated through lateral migration due to the asymmetric bifurcation of laminar flow [106]. Its microfluidic design features a main channel with multiple narrow channels branching at the end of pinch section, as showed in Figure 5(a). Such a channel design has resulted in the difference in flow velocity as well as particle volume fraction between the branches. Depending on the sizes and density of the particle, they will be pushed into specific flow streamlines following by separation. In the literature, this method is not much exploited in CTCs isolation. Geislinger and Franke are the first and only group who demonstrated continuous separation of cultured cancer cells (MV3-melanoma cell line) and blood cells using pinching flow as driving force [34]. The proposed microfluidic device worked at much lower Reynolds numbers (Re < 1) and an expansion with smooth broadening was added to increase the absolute distances to facilitate the isolation. The efficiency of 100% and medium purity of 66% are yielded in blood suspension with 9% of haematocrit. Although the result showed excellent cell viability, study has reported that such a technique is more suitable in a dilute suspension of blood sample. To improve the separation performance, this technique is normally integrated with dielectrophoresis and magnetophoresis method.

Apart of pinched flow fractionation, another promising candidate of hydrodynamic based separation is deterministic lateral displacement. This device is composed of an array of posts (see Figure 5(b)), in which each row is displaced at a distance, Δ*λ*, from the previous row. Similar to microfiltration, the dimension for both gap distance and obstacles size plays pivotal role in the separation process. When a given particle is smaller than the critical size, the particle will flow according to the mainstream line around the obstacle with nondeviating trajectory perpendicular to streamline. However, if the particle is bigger than the critical size, it will collide with the obstacle and results in streamline switch with a constant angle. Such a motion is termed as lateral displacement. By tuning the size of obstacle, the gap between them, and the shift in the array, particles from different size can be separated laterally. Using this technique, Davis et al. showed how the whole blood could be continuously separated into its constituents of erythrocytes, leukocytes, and plasma [105]. Despite its high sensitivity, this study has highlighted that the flow rate used in the device is relatively low compared to what would be necessary for rare cells sorting. To investigate the optimal flow rate for separation based on deterministic displacement, Loutherback et al. have demonstrated the isolation of CTCs from blood sample, within a long flow chamber filled with mirrored array of 58 *μ*m triangular posts with 42 *μ*m gaps. Their result presented a very good CTCs capture efficiency (>85%) at volumetric flow rate of 10 mL/min, with no effect on cell viability [22].

Inertial separation is another method which stands alone to manipulate particles in a continuous phase flow field. Contrary to both pinching flow fractionation and deterministic lateral displacement, this method dominates the fluid inertial lift force to accelerate the flowing particle to their preferential equilibrium points. Such a phenomenon can be observed in the spiral microchannel. The presence of curvature structure will generate rotational flow within the channel and thus result in the formation of two symmetrical counter-rotating vortices (top and bottom) across the channel cross section, termed as Dean vortices [106]. This motion entrains particle to move back and forth along the channel width. Coupled with inertial lift force, the larger particles (such as tumor cells) will occupy a single equilibrium position near the inner wall while the smaller particle (red blood cells and white blood cells) will migrate to the outer half of the channel, resulting in the formation of distinct particle streams which are collected in two separate outputs. Recently, a spiral device with 500 *μ*m wide and 160 *μ*m high using Dean flow induced motion was developed by Hou et al. for CTCs isolation purpose. When the device performance was tested with 3 mL blood suspension spiked with cancer cells (~20% hematocrit), it achieved high cancer cell recovery of >85% and high cell viability (>98%). Besides, followed by preliminary clinical testing, this device too had successfully detected CTCs in all blood samples collected from 20 patients with metastatic lung cancer, ranging from 5 to 88 CTCs per mL [107]. As opposed to Hou et al., Warkiani et al. investigated the performance separation within a spiral channel with trapezoidal cross section [35]. Their device had shown to achieve recovery rate of 80% for 7.5 mL of blood within 8 minutes. Similar to Hou et al., they also verified their method successfully being applicable to patient samples. However, such a device requires proper geometrical design. For instance, a deeper inner wall compared to the outer will cause strong vortices forces to be formed at the inner side of the channel, resulting in all particles to be trapped despite their different in size and flow rate [108]. Apart of spiral structure, Lee et al. had demonstrated their contraction-expansion array microchannel device (Figure 5(d)) to be able to sort different cancer cell lines out of the whole blood suspensions with a very high capture efficiency up to 99.5% [24, 109].

### 3.3. Summary of Device Performance

For benchtop CTCs' detection device, it is necessary to analyze and optimize the devices' performance before they are employed as clinical diagnostic tools. The key performance metrics which are widely highlighted in the literature included capture efficiency (or recovery rate), throughput, viability, and purity. Capture efficiency refers to the fraction of captured target cells relative to the total captured cells. It is usually expressed as a percentage (%). This measurement is important in accessing the total of CTCs which has been lost in the isolation process. A high capture efficiency represents less cell losses and thus provides clinician accurate information about the amount of CTCs discovered from patients' blood sample. Throughput indicates the speed at which the system can process a sample. In most experiments, high throughput device is favored by most researches as it allows a given sample to be analyzed within the short time This parameter is typically reported as either the volumetric flow or number of cells processed per second. Meanwhile, viability can be best described as the proportion of cells that remain “alive” and “viable.” This viability is commonly assayed by dye exclusion techniques where cells are incubated with a dilute solution of dye which only enters dead cells. Maintenance of cell viability is an imperative feature of cell separation procedure. For instance, the capture living cells can be used for downstream phenotypic and genotypic analyses. Furthermore, the cell behavior can be correlated with cell number, providing a more accurate picture of cell. Purity is defined as the percentage of cells in separated population that are detected as having certain design characteristics. It is an important indicator for the purpose of downstream analysis as a high purity indicates the cell subsets are not contaminated by nontarget cells. Subsequently, it will increase the sensitivity of assay used in postseparation analysis as well as provide accurate information of targeted cell. Purity assessment is typically conducted with a flow cytometer, in which target cells labelled with fluorescent markers are analyzed and the proportion of each cell type in sample is calculated.

## 4. Conclusion and Future Outlook

In this review paper, we have highlighted the development of several methods that exploit the physical characteristic of the cells to isolate and differentiate CTCs. Although these devices demonstrate significant progresses for CTCs isolation, the development of these technologies is still in diverging phase, in which methods described above are still at the proof of concept level. There are some challenges that remain for these devices to be fully employed in point-of-care application, including the usage of cultured cancer cell line, the used of whole blood sample, and low throughput.

Cultured cancer cell line is the most widely used in benchtop morphological model for CTCs, rarely yet on clinical samples. Its model is generated by isolating a tumor cell from a surgery sample and growing them in a controlled artificial environment. Unlike primary tumors which can only be maintained for a relatively short period of time in a reformulated culture system [110], cancer cell line is immortalized and genetically modified to proliferate indefinitely. Consequently, it gives rise to a clonal population and thus allows various scientific experiments to be carried out on these genetically identical cancer cells. In spite of their important contribution to cancer biology, several drawbacks are reported in the literature. Review written by both Okita and Yamanaka [111] and Holmberg and Perlmann [112] has indicated that tumor does not proliferate at the same rate as cultured cells. In fact, cultured cells are grown rapidly with doubling time much shorter than those of cancer cell in vivo. When cultured cancer cells are incubated in a growth promoting solution for a long period of time, they will induce occurrence of genomic changes, such as copy number variations as well as transcriptomic drift. This condition undoubtedly affects heterogeneity of cancer cell line and was proven in study conducted by Auman and McLeod [113]. Henceforth, the separation efficiency will be lower when the device is tested on the clinical sample, in contrast to the cancer cell line. To tackle this problem, effort should be made to develop new cell lines that exhibit the genomic and transcriptomic heterogeneity of cancer cell. Readers are encouraged to refer to Holmberg and Perlmann paper for more information [112].

As previously mentioned, CTCs can be detected in the bloodstream as early as before a primary tumor is detected with conventional clinical screening methods. As a result, complete blood analysis is of prime interest for most CTCs detection devices. Although the use of whole blood sample is preferable for device performance evaluation, fractioning various target components from it has been a technical challenge due to its massive number and wide diversity of cell type. Up to the moment, only two techniques (such as membrane microfilter device [15, 28, 29] and inertial separation device [24, 34]) are reported with high capture efficiency when the devices are tested with the whole blood samples (refer to Table 1). Majority of techniques have demonstrated a dramatic reduction in capture efficiency when the whole blood sample is employed. To circumvent this problem, the blood samples are generally diluted with isotonic diluent before proceeding to benchtop separation process. Dilution reduces the concentration of cell per unit volume and thus allows for rapid detection of certain particles from dense colonies. In fact, the presence of CTCs is extremely rare, 0 to 1 cells per millimeters of whole blood. Therefore, the use of dilution buffer will be able to decrease the number of CTCs within a sample, which in turn prolong the analysis time. Furthermore, study conducted by Takaori in 1979 has indicated that excessive dilution will cause a progressive decrease in blood sample pH. Since blood cells respond quickly to changes in microenvironment, their biological characteristic might change with regard to the reduction of pH. For instance, recent mathematical model developed by Wolf and DeLand has indicated that hematocrit varies with the change of blood pH [114]. In order to allow whole blood sample to be analyzed with bench-separation device, integration of multiple function such as enrichment and detection method onto single chips should be attempted. The enrichment technique (e.g., magnetophoresis) can be employed in first stage to increase the sensitivity of the assay by separating RBC from a human whole blood sample. This is followed by a detection step (e.g., dielectrophoresis or acoustophoresis) to separating target rare cells out from other nucleated cells and residual RBCs.

Multiples studies have suggested that the use of microfluidic devices are extremely attractive for blood analysis as these platforms allow miniaturization and integration of complex functions. As a result, the complete lab for blood analysis is feasible to be brought to patient's bedside. Besides, microfluidic revolves around the precise manipulation of fluid flow within channels, in which the dimension of the channel cross section is smaller than 1 mm^2^. Therefore, only minute amounts of blood are needed for analysis and repetitive sampling at multiple time points can be conducted. However, there is a drawback of using these devices, such that most of them are still rather limited in throughput. As shown in Table 1, the throughput is achievable by most microfluidic devices, lying around processing of 2 to 3 mL of blood per hours. Noteworthy, these devices are normally operated slowly to maintain separation efficiency. However, for diagnostic application, 7.5 mL of whole blood sample from patients is typically employed for CTCs enumeration. Henceforth, the flow rate required for optimum throughput is probably insufficient as it will take hours to complete the analysis. In this case, the separation time is increased, which leads to a loss of cell viability. Consequently, there is a need for researchers to develop a microfluidic architecture which allow for high throughput as well as enhancing the separation efficiency and viability of CTCs.

In fact, a few microfluidic techniques discussed in this paper has been commercialized for clinical diagnostic device to isolate live CTCs from epithelial and nonepithelial malignancies. These devices include ApoStream and ScreenCell.

**Figure 6:**
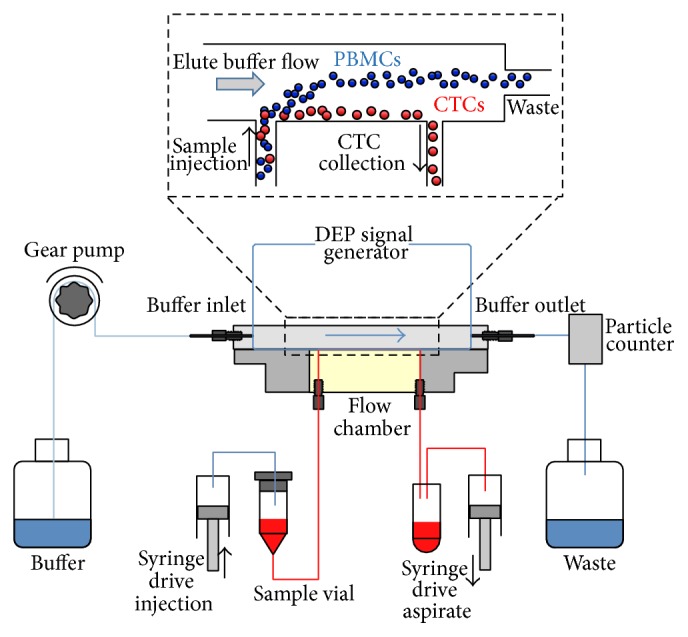
Illustration of ApoStream device. The elution buffer is introduced at the upstream end of the flow chamber with a computer-controlled gear pump. The blood sample is injected with a high precision syringe pump at a low flow rate into the bottom of the flow chamber to reduce the cell levitation and to ensure that cells stay within effective DEP field. Under DEP field, the DEP forces will attract cancer cells toward the electrodes on the chamber floors and vice versa to others cells. Cancer cells will withdraw through the collection port which is located close to the chamber floor. Meanwhile, other blood cells will be levitated and flow into the waste container via a second outlet port (reprinted with the permission from [25], copyright 2012, AIP Publishing LLC).

ApoStream system, as illustrated in Figure 6, has been launched by ApoCell laboratories in 2010 [115]. This technology leverages the dissimilarities of electrical properties of different cell types to isolate CTCs with the assistance of hydrodynamic force to position the cell at a defined level in a fluid velocity gradient. Interdigitated electrodes are fabricated on the chamber floor where the mixed cell population flows over the electrode structure. Since CTC population expresses positive DEP, it is pulled along the floor, flowing near the electrode plane and collected through a port located in the chamber floor, while the other cells (exhibiting negative DEP) are levitated and carried away by the eluent. Validation study of ApoStream system conducted by Gupta et al. using human blood sample spiked with breast cancer cell line (MDA-MB-231) has successfully demonstrated a cell viability of 97.6% with recovery efficiency of 86.6% [25]. In recognition of its potential in CTCs isolation, in December 2014, ApoStream is reported to be employed in BEACON (a Phase 3 open-label and multicenter study of Etirinotecan pegol versus treatment of physician's choice (TPC) in patients with metastatic breast cancer) to isolate CTCs for endpoint biomarker analysis [116].

The ScreenCell device is developed to support downstream analysis of captured CTCs, such as immunohistochemistry and nucleic acid isolation. This device consists of a filtration reservoir with a microporous membrane filter in a removable nozzle holder. The whole blood sample is needed to be diluted with standardized filtration buffer before it is placed on the filtration reservoir for 3 minutes sample processing time. To improve the throughput, vacuum is applied to the outflow side of the filtration membrane. Similar to the aforementioned membrane microfilter operating principle, CTCs will be retained on the surface of filtration membrane while small nucleated blood cell can easily pass through the filtration membrane. The membrane with captured CTC cells can be removed from the device in the end of filtration process for further manipulation. In 2011, ScreenCell study conducted by Desitter et al. on whole blood sample spiked with lung cancer cells has demonstrated a high recovery rate of 91% and 74% for 5 and 2 cancer cells, respectively, per mL of blood [117]. This device is further tested by Desitter group in enumeration of CTCs from 23 cutaneous melanoma patients (CTCs does not express EpCAM) for the purpose of cytological analysis and analysis of genetic mutations. The postsurgery median of CTC recovery is 1 CTC per 2 mL, with 15 of 23 patients being tested to be CTC-positive.

In conclusion, CTCs play an important role in both the research lab and the clinic. Due to their role in metastasis, information acquired from CTCs has the potential to assist in prognosis as well as helping to define individualized therapeutic regimens. While methods based on EpCAM enrichment have brought CTC to point of care application, Newer technologies which can facilitate CTC isolation as well as support downstream analysis techniques are in dire need. The features of user friendly and flexibility should be adapted into the CTC isolation device design consideration to make the technology available to more labs and clinic.

## Figures and Tables

**Table 1:** Performance specification of physical based CTCs separation methods.

Method	Target of cancer cell line	CTCs separation efficiency	Cell viability	Purity	Flow rate (whole blood/time)	References
Centrifugation (Ficoll-Hypaque)	Human breast cancer cells (MCF-7)	~46%	—	90%	—	Lara et al. [26]

Centrifugation (OncoQuick)	MCF-7, ZR-75-1, and Hs578T	MCF-7: 57.89% ZR-75-1: 59.80% Hs578T: 57.14%	—	—	—	Königsberg et al. [27]

Microfiltration (pore type)	Human breast cancer cells (MCF-7, MDA-MB-453, and SK-BR-3) and human prostate adenocarcinoma cancer cells (LNCAP, PC3)	7 *μ*m pore filter: (i) 98 ± 2% (fixed MCF-7 cells) (ii) 85 ± 3% (unfixed MCF-7 cells) 8 *μ*m pore filter: (i) 73 ± 13% (fixed MCF-7 cells) (ii) 50 ± 14% (unfixed MCF-7 cells)	—	—	~10 mL/min (concentration: 10^4^ cells/mL)	Adams et al. [10]
	Human prostate cancer PC-3 cells	>90%	>90%	—	—	Lu et al. [28]

Microfiltration (3D membrane)	Human prostate adenocarcinoma cell line (LNCaP) and human breast adenocarcinoma cell line (MCF-7)	>86%	85%	—	~3.75 mL/min (concentration: 10^6^ cells/mL)	Zheng et al. [11]
	Human gastric carcinoma cell line (NCI N-87)	>85%	—	—	2.5 mL/min (concentration: 2.5 × 10^5^ cells/mL)	Yusa et al. [12]
	Breast cancer cell lines: MCF-7, MDA-MB-231	78%–83%	71%–74%	—	— (1 *μ*/cancer cells spiked into 1 mL of undiluted blood)	Zhou et al. [13]

Microfiltration (weir-type)	Human A431 cancer cells	>95%	—	>98%	~5 mL/hr (10 tumors cells/mL of whole blood)	Chung et al. [15]

Microfiltration (pillar type)	MCF-7, SK-BR-3, J82, T24, RT4, and LNCaP	>90%	≥93%	>90%	—	Lin et al. [29]

Microfiltration (hetero-packed bead type)	Human breast cancer cells (MCF-7)	21%–40%	—		0.2 mL/hr (concentration: 10^6^/mL)	Arya et al. [30]

Dielectrophoresis (planar configuration)	Human breast cancer cells (MCF-7)	(i) MCF-7: 75.18% (ii) RBC: 99.24% (iii) WBC: 94.23%	—	(i) MCF-7: 16.24% (ii) RBC: — (iii) WBC: —	~250 *μ*L/s	Moon et al. [31]

Dielectrophoresis (contactless mode)	Human cervical carcinoma cell (HeLa cells)	64.5%	—	—	— (concentration in diluted solution: 2.5 × 10^5^ HeLa cells/mL, 3.25 × 10^6^ RBC cells/mL, 3.25 × 10^3^ WBC cells/mL)	Huang et al. [19]

Acoustophoresis	Prostate cancer cell line:					
	DU145, PC3, and LNCAP	PFA-fixed cell sample (i) DU145 and PC3: 85.4% at *E*_ac_ = 120 J/m^3^, 96.6% at *E*_ac_ = 188 J/m^3^ (ii) LNCaP: 55.5% at *E*_ac_ = 120 J/m^3^, 78.8% at *E*_ac_ = 188 J/m^3^ Non-PFA-fixed cell sample (i) DU145: 36.1% at *E*_ac_ = 66 J/m^3^, 83.7% at *E*_ac_ = 103 J/m^3^	No significant changes were observed for acoustophoresis treated and untreated samples	PFA-fixed cell sample (i) DU145 and PC3: 99.3% at *E*_ac_ = 120 J/m^3^, 97.9% at *E*_ac_ = 188 J/m^3^ Non-PFA-fixed cell sample (i) DU145: 99.5% at *E*_ac_ = 66 J/m^3^, 93% at *E*_ac_ = 103 J/m^3^	~450 *μ*L/min (2.5 × 10^5^ tumor cells/mL spiked with 10-fold diluted whole blood)	Augustsson et al. [32]
	DU145, PC3, LNCAP, and VCaP	—	The average cell dead is <1% for DU145 and PC3, while ≤3% for LNCAP and VCaP	—	~100 *μ*L/min	Burguillos et al. [33]

Hydrodynamic sorting (pinched flow fractionation)	MV3-melanoma cells	100%	~100%	(i) 0.4% hematocrit: 66.6% (ii) 4% hematocrit: 15.4% (iii) 9% hematocrit: 5.5%	600 *μ*L/h (concentration in diluted solution: 8 × 10^5^ tumor cells/mL, 6 × 10^8^ RBC cells/mL)	Geislinger and Franke [34]

Hydrodynamic sorting (deterministic lateral displacement)	Human breast cancer cell line: MDAMB231, MCF10A, and PC3	≥85%	≥95%	—	10 mL/min (concentration: 10^7^ cancer cells in 1 mL of blood diluted with buffer)	Loutherback et al. [22]

Hydrodynamic sorting (inertial lift force)	MCF-7	>85%	>98%	50%	3 mL/hr (concentration: 10–100 CTCs/mL of diluted whole blood samples)	Hou et al. [23]

Hydrodynamic sorting (inertia force)	MCF-7, T24, and MDA-MB-231	>80%	>80%	~4 log depletion of WBCs	2.0 mL/min (500 tumour cells per 7.5 mL of whole blood)	Warkiani et al. [35]

Hydrodynamic sorting (inertia force)	MCF-7 cancer cells	99.5%	—	—	3 to 12 mL/h (processing 4.2 × 10^7^ cells/min to 2 × 10^7^ cells/min)	Lee et al. [24]
